# Recent Advances on Endocrine Disrupting Effects of UV Filters

**DOI:** 10.3390/ijerph13080782

**Published:** 2016-08-03

**Authors:** Jiaying Wang, Liumeng Pan, Shenggan Wu, Liping Lu, Yiwen Xu, Yanye Zhu, Ming Guo, Shulin Zhuang

**Affiliations:** 1 Institute of Environmental Science, College of Environmental and Resource Sciences, Zhejiang University, Hangzhou 310058, China; 21514005@zju.edu.cn (J.W.); panliumeng@zju.edu.cn (L.P.); lulipingelma@163.com (L.L.); xyw304@163.com (Y.X.); 3140102658@zju.edu.cn (Y.Z.); 2 Key Laboratory of Health Risk Factors for Seafood of Zhejiang Province, Zhoushan 316022, China; 3 Institute of Quality Standard of Agro-Products, Zhejiang Academy of Agricultural Sciences, Hangzhou 310021, China; wushengggan@163.com; 4 School of Science, Zhejiang Agriculture & Forestry University, Lin’an 311300, China; guoming@zafu.edu.cn; 5 Guangzhou Key Laboratory of Environmental Exposure and Health, School of Environment, Jinan University, Guangzhou 510632, China

**Keywords:** ultraviolet filters, cosmetics, endocrine disrupting effects, nuclear receptor

## Abstract

Ultraviolet (UV) filters are used widely in cosmetics, plastics, adhesives and other industrial products to protect human skin or products against direct exposure to deleterious UV radiation. With growing usage and mis-disposition of UV filters, they currently represent a new class of contaminants of emerging concern with increasingly reported adverse effects to humans and other organisms. Exposure to UV filters induce various endocrine disrupting effects, as revealed by increasing number of toxicological studies performed in recent years. It is necessary to compile a systematic review on the current research status on endocrine disrupting effects of UV filters toward different organisms. We therefore summarized the recent advances on the evaluation of the potential endocrine disruptors and the mechanism of toxicity for many kinds of UV filters such as benzophenones, camphor derivatives and cinnamate derivatives.

## 1. Introduction

Ultraviolet (UV) filters are a class of chemicals that can absorb or reflect UV light in the ultraviolet A (UVA) range and ultraviolet B (UVB) range with specific wavelengths between 320 and 400 nm, 290 and 320 nm, respectively [1]. They can protect human skin against direct exposure to deleterious UV radiation [2,3]. Many kinds of organic UV filters were incorporated into cosmetics, plastics, adhesives and other industrial products to avoid potential UV-induced damage. There are 16 UV filters permitted to be used in cosmetics by the US Food and Drug Administration and 27 components permitted to be used in cosmetics by the EU Scientific Committee on Consumer Products [4].

Many UV filters were produced in large quantities and used widely [5]. Residues of UV filters have been detected in multiple environmental matrices including wastewater treatment plants, surface water, sewage sludge, river sediments, fish, human milk and placenta [6,7,8,9]. UV filters can be bioaccumulated in organisms due to their persistence, stability and lipophilicity [10,11]. They are now becoming contaminants of emerging concern [12,13,14]. UV filters were reported to induce acute toxicities, developmental toxicities and reproductive toxicities to different organisms [15,16,17,18,19,20,21].

Many kinds of UV filters (Table 1) have been identified as potential environmental endocrine disruptors [22]. There are an increasing number of studies on endocrine disrupting effects of UV filters. Exposure to UV filters was increasingly reported to cause the disruption of the endocrine systems in many organisms such as rat, frog *Xenopus laevis*, Japanese quail (*Coturnix japonica*), *Chironomus riparius* (Meigen) and fish [22,23,24,25,26,27,28,29,30,31]. Considering increasing toxicological studies performed in recent years, a systematic compilation of current research status on their endocrine disrupting effects is necessary. We therefore compiled a state-of-the-art review on the endocrine disrupting effects of many type of UV filters (Table 2).

## 2. Endocrine Disrupting Effects of Typical UV Filters

### 2.1. Benzophenones

Benzophenone (BP)-type UV filters were used widely in many cosmetics for the protection of skin from UVA and UVB light [12]. Their molecular structures have a diarylketone scaffold with different substitute groups. Residues of BPs were detected in wastewater, surface water, soil, sediment, human urine and breast milk [55,56,57,58]. Many BPs were identified as endocrine disruptors and were involved in the disruption of the hypothalamic–pituitary–gonadal system [13,59]. As revealed by various in vivo, in vitro bioassays and in silico methods, BPs showed multiple endocrine disrupting effects toward estrogen receptor (ER), androgen receptor (AR), progesterone receptor (PR) and other nuclear receptors [22,32,38,39,60].

#### 2.1.1. Estrogenic Disrupting Effects

BP-type UV filters could cause multiple estrogenic effects, developmental and reproductive toxicity as revealed by cell-based bioassay [26,33,34,43,61]. BP-3 was reported to cause a dose-dependent increase of uterine weight of immature Long-Evans rats by the activation of ERα and ERβ [22,43]. BP-2 caused estrogenic effects such as vitellogenin (VTG) induction in fish [35]. BPs such as BP-1, BP-2, BP-3 and BP-4 caused estrogenic activity, developmental and reproductive toxicity to fish and rat [26,35,36,43]. BPs showed moderate potency to activate the proliferation of MCF-7 breast cancer cell and estrogen-responsive CHO cells at concentrations of the order of micromolar and lower [37,62]. This potency ranks as 4-hydroxy-benzophenone > 4,4′-dihydroxy-benzophenone > BP-8 > 2,3,4,4′-tetrahydroxy-benzophenone > BP-2 > 2,4, 4′-trihydroxy-benzophenone. However, there was no significant induction of proliferation induced by BP, BP-1, BP-3 and 2,3,4-trihydroxy-benzophenone. Molecular modeling can provide atomic-level information and has been well used to probe interactions of chemicals with biomacromolecules [39,63,64,65,66]. BPs interacted with residues Glu353, Arg394 or Phe404 of ERα ligand binding domain and such binding mode enhanced binding stability, contributing partly to their estrogenic activities [62].

Biotransformation or chemical transformation of BPs may have influence on their endocrine disrupting effects. BP-3 can be metabolized within human body and can be metabolized to various metabolites including BP-1 and BP-3. BP-1 was detected in human urine [37] and BP-1 and BP-8 were detected in rats [67,68]. BP-1 possessed higher estrogenic activity than that of BP-3 [32,33,34]. BPs were revealed to be converted to 4-hydroxybenzophenone after exposure to sunlight, indicating the potential estrogenic risk of BP-containing sunscreen in direct contact with the skin [62,69].

#### 2.1.2. Androgenic Disrupting Effects

BP-1, BP-2 and BP-3 showed no agonistic activity toward AR [50]; however, they exhibited anti-androgenic activity in various cells-based bioassays [33,34,36,38]. BP-1, BP-2 and BP-3 showed complete inhibition of dihydrotestosterone activity in concentration-dependent mode. BPs disturbed the normal hormonal level of testosterone during male development of mouse and rat by inhibiting the conversion of androstenedione to testosterone [39,70]. BP-2 displayed antagonistic activity with non-monotonic dose–response curves. There were very weak or no inhibitory effects for BP-4, BP-7, BP-8 and BP-12 and weak effects for BP-2, BP-3 and BP-6 [39]. The androgenic disrupting effects of BPs were also affected by the biotransformation of BPs. BP-3 showed decreased androgenic activity after the metabolism mediated by rat and human liver microsomes [37]. BP-1 was the most potent anti-androgenic UV filter and concentration dependently inhibited 17β-HSD3.

#### 2.1.3. Disrupting Effects toward Other Nuclear Receptors

BPs also exhibited disrupting effects towards PR and thyroid hormone receptor (THR). BP-3 exhibited antagonistic effects to PR and BP-2 interferes with the thyroid hormone (TH) axis in rats [38,40]. Although BP-3 did not activate PR, it is the antagonist of PR as revealed by PR CALUX1 bioassay [38]. BPs can also affect the TH axis by inhibiting the activity of thyroid peroxidase (TPO) or inactivate it, disturbing the biosynthesis of TH [71]. BP-2 was demonstrated as a very potent inhibitor of TPO activity [41]. The study revealed that BP-2-treated rats exhibited decreased thyroxine (T4) and increased thyroid-stimulating hormone (TSH) serum levels. BP-2 disturbed TH homeostasis by inhibiting or inactivating TPO as revealed by the stably transfected human recombinant TPO [40]. As evaluated by an in vitro reporter system containing a duplicated thyroid hormone response element of the HLA-DR4 serotype, BP-2 and BP-3 can induce luciferase activity, showing agonistic activity toward THR [42].

### 2.2. Camphor Derivatives

Camphor derivatives are highly effective UVB-absorbers incorporated in many kinds of cosmetics. These chemicals have high bioconcentration factors and can be bioaccumulated in tissues of organisms after prolonged exposure [72,73]. The camphor derivatives such as 4-methylbenzylidene camphor (4-MBC) and 3-benzylidene camphor (3-BC) are very lipophilic and can be easily absorbed after direct contact with the skin. 4-MBC and 3-BC were revealed as potential endocrine disruptors, adversely affecting the reproduction and development of many organisms [74,75].

#### 2.2.1. Disrupting Effects toward Estrogen Receptor

4-MBC and 3-BC showed anti-estrogenic activity in fish, mammals and cell-based bioassays [22,44,76]. Exposure to 3-BC during the early development and postnatal life of rat, could lead to significant changes of the expression of ERs and estrogen target genes [77]. 4-MBC and 3-BC also showed specific estrogenic activity in the HELN ERα cell and MCF-7 cell line proliferation assay [22,45]. 3-BC exhibited estrogenic potency in immature rats [76] and also showed high estrogenic potency of inducing VTG in juvenile fathead minnow [26]. 4-MBC and 3-BC negatively affected the sex ratio of frog *Xenopus laevis* at environmental concentrations [23]. They were selective ER ligands [46] and bound preferentially to ERβ [76]. 4-MBC activated ERα weakly and showed higher potency toward ERβ mediated transactivation in Ishikawa cells [47]. 4-MBC could activate human ERα in concentration-dependent mode and also significantly activate transcription through human ERβ [43]. 4-MBC caused an increase of mRNA expression level of ERα and VTG in male Japanese medaka [48].

#### 2.2.2. Disrupting Effects toward Androgen Receptor

4-MBC and 3-BC showed no agonistic activation toward AR [50]. They exhibited anti-androgenic activity toward AR in AR CALUX^®^ cell line as revealed by the transcriptional-activation assay [38,50]. They could inhibit the activity of AR in a concentration-dependent mode as revealed by the recombinant yeast assays [36]. 4-MBC was proved to be a potent human AR antagonist and significantly inhibited luciferase activity [78]. 4-MBC and 3-BC also concentration-dependently prevented testosterone formation by inhibiting androgen-metabolizing 17β-HSD3 in HEK-293 cells [39].

#### 2.2.3. Disrupting Effects toward Progesterone Receptor

The developmental 4-MBC exposure could cause an increase of PR mRNA levels in male medial preoptic area, but this change was not detected in female rat [52]. 4-MBC at very low dose can down-regulate the expression level of PR protein. With the increasing doses, the expression of PR protein returned to normal or slightly supranormal levels [77]. 4-MBC disturbed the expression of membrane-associate PR, measured by changes in mRNA levels at different developmental stages [51]. 4-MBC and 3-BC showed no PR transactivation in U2-OS cells, and these two UV filters at low concentrations were antagonists of PR [38].

### 2.3. Cinnamate Derivatives

Cinnamate derivatives are the most frequently used cosmetic UV filters with the high efficiency to absorb UVA or UVB light. Their molecular structures have a special unsaturated bond between the aromatic ring and the carboxyl group, allowing the molecule to better absorb the 305 nm wavelength UV [79]. Their residues were detected in wastewater, surface water, sewage sludge, fish and marine mammals [11,80,81]. Octyl methoxycinnamate (OMC) is one of the most commonly-used UV filters. It was listed as one of 27 UV filters approved for use in cosmetics formulations in the EU and US [82]. OMC has disrupting activities toward ER, AR, PR and THR as reported by multiple in vitro and in vivo studies [16,38,78].

#### 2.3.1. Disrupting Effects toward Estrogen Receptor

The potential estrogenic activities of cinnamate derivatives have been reported by various in vivo and in vitro experiments [16,22,43]. OMC, isopentyl-4-methoxycinnamate (IMC) and octocrylene (OC) could completely inhibit the activity of E2 by yeast human assays [36]. OMC was found to moderately activate ERα but no obvious effect on ERβ by using reporter cell lines including HELN, HELN ERα, and HELN ERβ [45] was observed, in line with the transactivation bioassay using HEK293 cells in which OMC dose-dependently activated ERα, but did not activate transcription of ERβ [43]. Exposure to OMC induced weak estrogenic effect to the uterus and the vagina of female Sprague–Dawley rats [49]. OMC could cause an increase of plasma concentration of VTG and up-regulate the mRNA expression levels of ERα in medaka fish [48].

#### 2.3.2. Disrupting Effects toward Thyroid Hormone Receptor

Cinnamate derivatives also interfered with the TH axis in rats. The perinatal OMC-exposure could induce adverse effects on the reproductive and neurological development of rat offspring [16,53]. The treatment with OMC for 12 weeks caused a decrease of T4 level in the blood of ovariectomised female rats and inhibited the activity of 5′-deiodinase that converts T4 to T3 in the liver [53]. Exposure to OMC caused a dose-dependent decrease of serum concentrations of TSH, T4 or T3 in rats [54]. TPO activity was unaltered but T3-responsive hepatic type I 5′ deiodinase activity was reduced by OMC. OMC was found exerting effects on the TH axis by using female ovariectomized rats [83]. OMC affected TH via inhibition of type I 5′-Deiodinase activity and gene expression.

#### 2.3.3. Disrupting Effects toward Other Nuclear Receptors

Cinnamate derivatives also induced disrupting effects toward other NRs such as PR and AR. OMC, IMC and OC showed obvious antagonistic effects toward PR and AR as revealed by cell-based bioassays and in vivo experiments using rats [16,38]. OMC showed antagonistic activity toward PR as determined by sensitive and specific reporter gene cell lines [38]. OMC, IMC and OC showed anti-androgenic activities as revealed by the yeast assay [36]. They inhibited 4,5-dihydrotestosterone activity in concentration-dependent mode.

## 3. Perspectives

With the continuous demand of cosmetics, plastics and various industrial products containing UV filters, the production and application of UV filters, especially new type of UV filters will be increased. For better risk assessment of these chemicals and their metabolites, the investigation of endocrine disrupting effects caused by direct and indirect exposure to UV filters is currently becoming a research hotspot. Adverse outcome pathways of emerging UV filters should be well characterized for their potential disruption of the hypothalamus-pituitary-gonadal endocrine axis. Although multiple in vivo and in vitro studies have investigated the adverse effects of UV filters, the underlying mechanism of endocrine disruption should be further explored at the atomic level. Considering the versatile role of computational toxicology for the study of physiochemical properties of organic contaminants and their interactions with various biomacromolecules [39,63,84,85], more in silico studies should be performed, primarily for UV filters to probe the molecular initiating event toward target receptors.

## 4. Conclusions

We reviewed the potential endocrine disruptors of typical UV filters including benzophenones, camphor derivatives and cinnamate derivatives. These UV filters are generally involved in the disruption of the hypothalamic–pituitary–gonadal system. As revealed by in vivo and in vitro assays, exposure to these chemicals induced various endocrine disrupting effects such as estrogenic disrupting effects, androgenic disrupting effects as well as the disrupting effects towards TR, PR. The underlying mechanism of endocrine disruption was summarized (Table 2). The minor structural changes of these kinds of UV filters have influence on the potency of their endocrine disrupting effects.

## Figures and Tables

**Table 1:** The commonly used ultraviolet (UV) filters.

Compound	CAS No.	Chemical Structure	Kp (cm/h) *
BP-1	131-56-6	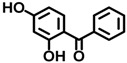	0.00917
BP-2	131-55-5	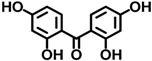	0.00458
BP-3	131-57-7	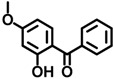	0.0271
BP-4	4065-45-6	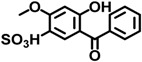	0.0000511
4-MBC	36861-47-9	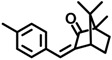	0.504
3-BC	15087-24-8	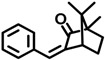	0.261
OMC	5466-77-3	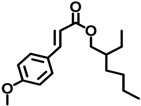	0.264
IMC	71617-10-2	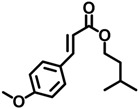	0.0477
OC	6197-30-4	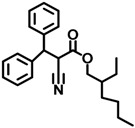	0.549

BP: benzophenone; 4-MBC: 4-methyl benzylidene camphor; 3-BC: 3-benzylidene camphor; OMC: octyl methoxycinnamate; IMC: isopentyl-4-methoxycinnamate; OC: octocrylene. * Kp, the dermal permeability coefficient, calculated by Program (DERMWIN) v2.0, was calculated following the equation: Log Kp = −2.80 + 0.66 Log Kow − 0.0056 MW.

**Table 2:** Endocrine disrupting effects of the commonly used UV filters.

UV Filters	Endocrine Disrupting Effects		References
**Benzophenones**	**Estrogenic disrupting effects**	Activation of ERα, ERβ; Inhibition of the activity of 17β-Estradiol; Induction of proliferation of MCF-7 cell; Induction of VTG in fathead minnows; Reduce of the uterine weight in immature Long-Evans rats	[22,26,32,33,34,35,36,37]
	**Androgenic disrupting effects**	Antagonists of human AR transactivation; Repression of 4,5-dihydrotestosterone-induced transactivational activity; Inhibition of testosterone formation in mice and rats	[34,36,37,38,39]
	**Disrupting effects toward other nuclear receptors**	Inhibition of human recombinant TPO; Interference with THR; Inhibition of TPO activity in rats; Antagonists of PR	[38,40,41,42]
**Camphor derivatives**	**Disrupting effects toward estrogen receptor**	Activation of ERα, ERβ; Inhibition of the activity of 17β-Estradiol; Induction of proliferation of MCF-7 cell; Induction of pS2 protein in MCF-7 cells; Reduce of the uterine weight in rats; Induction of VTG in fish	[22,26,43,44,45,46,47,48,49]
	**Disrupting effects toward androgen receptor**	Repression of 4,5-dihydrotestosterone-induced transactivational activity; Inhibition of testosterone formation in HEK-293 cells; Antagonists of Human AR	[36,38,39,50]
	**Disrupting effects toward progesterone receptor**	Antagonists of PR; Increase of PR mRNA levels in rats; Inhibition of the expression of PR protein in rats; Disturbance of the expression of membrane-associate PR in insects	[38,47,51,52]
**Cinnamate derivatives**	**Disrupting effects toward estrogen receptor**	Activation of ERα; Inhibition of the activity of 17β-Estradiol; Induction of proliferation of MCF-7 cell; Reduce of the uterine weight in rats; Induction of VTG in fish	[22,36,43,45,48,49]
	**Disrupting effects toward thyroid hormone receptor**	Decrease of T4 level; Inhibition of the conversion of T4 to triiodothyronine in rats	[16,53,54]
	**Disrupting effects toward other nuclear receptors**	Antagonists of PR and AR; Inhibition of 4,5-dihydrotestosterone activity; Reduce of the prostate and testicular weight in rats	[16,36,38]

AR: androgen receptor; ER: estrogen receptor alpha; PR: progesterone receptor; T4: thyroxine; THR: thyroid hormone receptor; TPO: thyroid peroxidase; VTG: vitellogenin.
